# Endocrine Changes in Postmenopausal Women: A Comprehensive View

**DOI:** 10.7759/cureus.51287

**Published:** 2023-12-29

**Authors:** Vidhi Motlani, Gunjan Motlani, Soumya Pamnani, Akshat Sahu, Neema Acharya

**Affiliations:** 1 Department of Obstetrics and Gynaecology, Jawaharlal Nehru Medical College, Datta Meghe Institute of Higher Education & Research, Wardha, IND

**Keywords:** hormone therapy, vasomotor symptom, metabolic syndrome, cardiovascular disease, menopause symptoms

## Abstract

Menopause, when menstrual cycles stop, is brought on by a decline in the level of the hormones progesterone and oestrogen synthesised by the ovaries. Menopause is an unavoidable stage of a female's lifecycle, but because experiences differ for every woman, several women require health care aid to manage their health problems. The physiological variations that take place at various periods of the reproducing age, along with the kind and timing of menopause, are components that are frequently associated with a greater threat of cardiometabolic illness. The most researched associations between menopause and cardiometabolic health are reduced levels of ovarian estrogen synthesis and excessive amounts of androgen during the onset of menopause. Although testosterone and oestrogens have differing effects on adipocyte physiology, it is debatable how important oestrogens are for the emergence of metabolic disorders following menopause. The control of adipocyte differentiation by the brain as well as potential roles of oestrogen and endocrine disruptors chemicals are reviewed in this systematic review of the subject. In general, women had a greater frequency of metabolic syndrome compared to men. Female metabolism was significantly impacted by overt hyperthyroidism and subclinical hypothyroidism. Osteoporosis is another medical condition that menopausal women may experience. Estrogen deprivation is the main contributor to osteoporosis in menopausal women. The regular cycle of bone turnover is disrupted by the decrease in estrogen secretion, which boosts osteoclastic resorption activity while decreasing osteoblastic activity. The entire article assesses and provides information on all the changes in a woman's life after menopause.

## Introduction and background

Menopause is characterised by the permanent cessation of cycles of menstruation, which are brought on by a reduction in the amount of progesterone and estrogen from the ovaries. Even though menopause is a natural occurrence for females, personal encounters might differ. Menopause has been linked to a number of manifestations, although only vasomotor disorder and dryness of the vagina are reliably connected with the condition and some women take medication to manage symptoms. Assessments of therapeutics for vasomotor impairment have demonstrated benefits with clonidine, paroxetine, gabapentin, and oestrogen [1]. A woman is diagnosed with menopause when she has not menstruated for over one year due to the cessation of follicular activity in ovaries, which naturally occurs in the majority of women between the fifth and sixth decade of life and women experience accelerated physical, physiological, and neuroendocrine ageing during menopause. The constellation of adverse changes that result from the altered hormonal environment of menopause, particularly the abrupt decline in oestrogens that accompany this transition, dramatically increases the risk of cardiovascular disease [2]. Menopause can be caused by follicular depletion, which is natural menopause, or it can be iatrogenic or induced by bilateral oophorectomy or radiation exposure to the ovaries [3]. Every year, millions of women go through the menopause transition, which is regularly associated with bothersome symptoms like vasomotor symptoms, dryness of the vagina, decreased libido, sleep disturbances, exhaustion, and joint pain [4].

Endocrine changes which start around menopause and sudden loss of the release of hormones have an impact on numerous bodily systems. Menopause symptoms involve cardiovascular, musculoskeletal, metabolic, and weight abnormalities, as well as genitourinary and epidermal degeneration and hirsuteness, along with central nervous system ailments [5,6]. Though the biological explanation for these manifestations is complicated and linked, estrogen insufficiency isn't the only reason. In addition, metabolism-related issues are strongly linked, persistent, and advancing, and they have a significant impact on the general well-being of postmenopausal females [7]. The rise in the average lifespan, particularly among women, extends the period a woman has after menopause while also raising consciousness of its influence on numerous health concerns throughout the postmenopausal era [8,9]. 

Alterations in hormones are among the most significant physiological alterations linked with menopause. Estradiol, a primary sexual hormone in females, governs secondary sexual traits and impacts the growth and functionality of the genital system in females. The physiological and metabolic changes linked to menopause are ascribed to a decreased oestrogen level. This hormonal deficiency has been observed to impact various aspects of the metabolism of lipids in the body, energy consumption, insulin sensitivity, and body adipose tissue distribution [10]. Specifically, there is a transformation from a gynoid body shape to an android body shape, along with increasing subcutaneous and belly fat deposition. These alterations in the body are associated with a greater likelihood of metabolic and cardiovascular complications. Metabolic syndrome (MetS) encompasses a cluster of many variables, like hypertension, dyslipidemia, insulin resistance, obesity, and glucose intolerance. These factors collectively elevate an individual's susceptibility to the progression of cardiovascular disease (CVD) and type 2 diabetes [11]. The menopausal transition, sometimes referred to as perimenopause, denotes the onset of monthly irregularities accompanied by symptoms indicative of a shortage in female sex hormones [12]. We researched the metabolic abnormalities, particularly MetS, believed to emerge in postmenopausal women. We established their genesis, diagnoses, and management approaches to decide which strategy is required for optimal well-being in postmenopausal women.

## Review

Methodology

**Figure 1:**
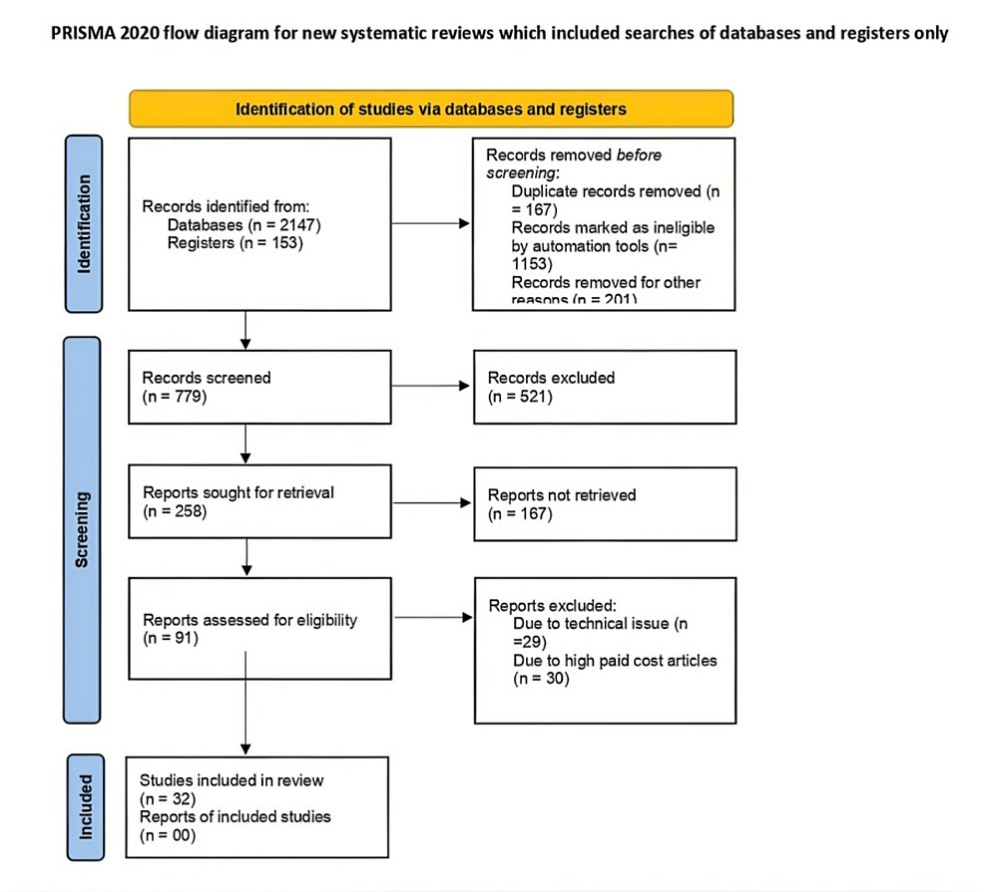
PRISMA flow diagram for literature search PRISMA: Preferred Reporting Items for Systematic Reviews and Meta-Analyses

The terms like “menopause”, “metabolic syndrome”, “cardiovascular disorder”, “diabetes mellitus”, and “hormone therapy” were searched in the PubMed database. The latest articles were selected for reference. Only review articles were taken into account. The inclusion criteria included published articles in the English language containing the above-mentioned keywords from 2005 to July 2023. We excluded studies conducted before 2005 and studies in languages other than English. We also excluded studies whose full text was unavailable to us due to resource limitations. We also excluded studies which focused on subjects on hormone replacement therapy for any reason (Figure 1). 

Pathophysiology of menopause 

The pathophysiology of the menopausal transition is complicated, and the circulation of blood levels of oestradiol, follicle-stimulating hormone (FSH), and luteinising hormone (LH) can change significantly in the initial phases of the transition. Regular hormone screenings are not required in women over the age of 45 with conventional signs and symptoms for confirmation of menopause and the possibility of initiating hormone replacement medication [13]. When the quantity of oocytes reaches an essential number and ovarian follicular development ceases, the feedback process within the ovary, pituitary, and brain is engaged, producing variations in gonadotropins [14]. Follicular stage inhibin B hormone levels decline while FSH levels rise. The altering hormones are related to anovulatory menstrual cycles that proceed until the last menstrual bleeding. Increasing gonadotropins keeps serum oestradiol concentrations stable until late throughout the menopausal transition. Such hormonal fluctuations often result in erratic menstruation cycles, with shorter cycle lengths in the early phases and gradually longer intervals between periods as time passes. Circulating levels of testosterone do not fluctuate much throughout the early menopausal transition, which alters the ratio of androgens to oestrogens, causing signs of androgen surplus in certain women [14]. 

**Figure 2:**
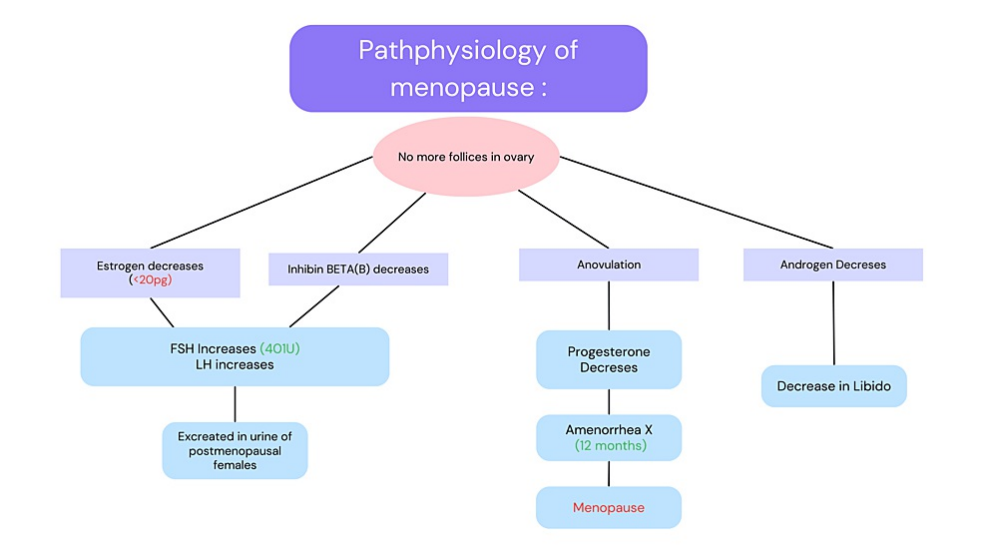
Pathophysiology of menopause Figure Credit: Author Vidhi Motlani FSH: follicle-stimulating hormone; LH: luteinising hormone

Every month, follicular stage FSH promotes follicular development and estradiol production in menstrual women. Increased estradiol synthesis by the prevailing follicle has a negative feedback and inhibiting impact on FSH and LH. Estradiol production by the predominant follicle persists till an appropriate amount is achieved, at which point positive feedback, an LH surge, and ovulation occur [15]. Multiple physiologic changes during the menopausal transition result from reduced ovarian reserve and reduced numbers of gonadotropin-responsive follicles. Menstrual cycles in late perimenopausal women are characterized by increased FSH, decreased inhibin B (Figure 2), and irregularly short and long cycle lengths [16]. Estradiol levels of menopausal women vary similarly till the last menstrual period (LMP) [17,18]. Women develop a permanent condition of hypogonadism and hypergonadotropism (elevated FSH and LH) by the LMP. Estrone, predominantly produced after the aromatization of androgens, replaces estradiol as the primary systemic estrogen. In comparison to estradiol, serum levels of testosterone show a slower yet more consistent fall [19,20]. The comparatively less significant drop in blood androgenic hormones is due to a reduction in sex hormone-binding globulin, which correlates with hypoestrogenism [21]. 

Hormonal changes in menopause

Hormonal alterations are widely recognised as a prominent physiological consequence of menopause. There exist three distinct forms of estrogen, namely estrone (E1), estradiol (E2), and estriol (E3). They have all been classed as 18-carbon (C18) steroids and possess aromatic molecular structures [22]. Each kind of contraception possesses a distinct purpose that aligns with the many stages of the life cycle of a woman, encompassing reproductive age, pregnancy, and menopause. Meningeal cells produce androstenedione during the perimenopausal stage and the menstrual cycle. In the ovaries and other peripheral organs, this hormone functions as a metabolic precursor of E1 and testosterone. Within the granulosa cells, the enzymatic activity of cytochrome P450 family 19 (CYP 19), specifically aromatase, facilitates the conversion of androstenedione to E1. Subsequently, E3 undergoes conversion to E2 [23]. The collective concentration of estrogen, including E1, E2, and E3, typically ranges from 100 to 250 picograms per millilitre. In contrast, it is seen that circulating levels of E2 significantly decrease to approximately 10 pg/mL in postmenopausal women. A few years before menopause, inhibins A and B drop in levels, plasma estradiol < 20 pg/ml (range 5-25 pg/ml), and elevated FSH >50 mU/ml are in sync with the ovarian function cessation. Consequently, it can be inferred that women have an oestrogen shortage for approximately half of their lifespan [23,24].

Symptoms of menopause in pre- and postmenopausal women

**Figure 3:**
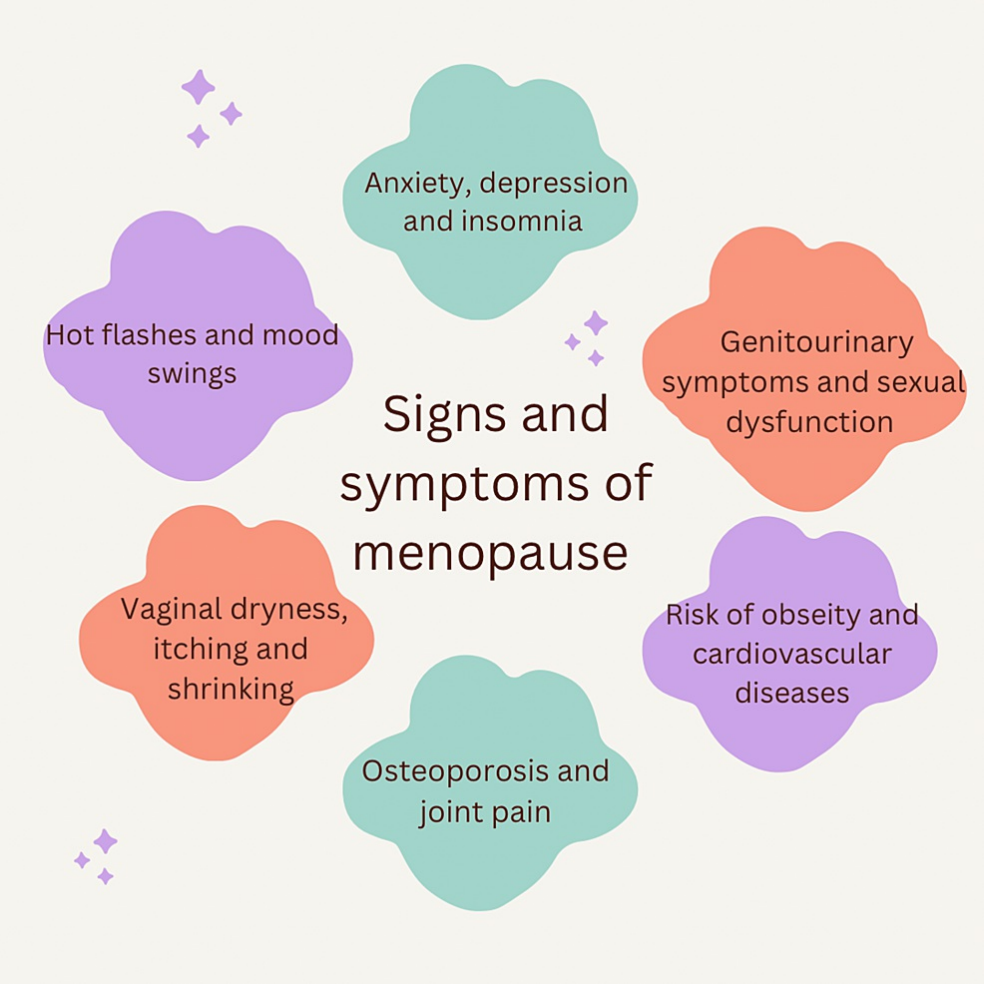
Signs and symptoms of menopause Figure Credit: Author Vidhi Motlani

Menopause is a physical process. The natural menopause may be genetically determined in healthy women. The annual incidence of the menopause transition affects approximately 1.5 million women. Although endocrine changes are permanent, menopausal symptoms such as hot flashes, which are experienced by approximately 70% of women, typically subside with time [11]. However, they can persist for decades in a minority of women [25]. During the menopausal transition, women may notice a few symptoms like menstrual irregularities, vasomotor alterations, libidinal decline, urinary difficulties, emotional swings, and cognitive decline. Nevertheless, specific symptoms, such as genital atrophy, may persist or aggravate. The changes associated with declining ovarian function, especially the rapid decline in oestrogens that defines this transition, increase the chance of heart disease due to a confluence of factors [26]. It is frequently accompanied by distressing manifestations like vasomotor impairment, dryness of the vagina, reduced libido, disturbed sleep patterns, exhaustion, and musculoskeletal discomfort (Figure 3) [27]. This hormonal deficiency has been observed to impact various aspects of the metabolism of lipids in the body, energy consumption, sensitivity to insulin, and distribution of adipose tissue. These alterations are linked to an increased cardiovascular and metabolic disease risk and early prevention, detection, and management will be aided by an increased awareness of the nature of risk factors associated with these typical symptoms in menopausal women [1].

MetS in menopause

The incidence of MetS is increasing on a worldwide scale. MetS refers to a collection of many variables, which include hypertension, dyslipidemia, cellular resistance to insulin, obesity, and glucose intolerance. These factors collectively elevate individuals' susceptibility to developing cardiovascular disease and type 2 diabetes [28]. The prevalence of MetS is increasing on a global scale. The menopausal transition, referred to as perimenopause, denotes the onset of monthly irregularities accompanied by symptoms indicative of a shortage in female sex hormones [10]. Menopause is linked to alterations in adipose tissue's morphology and quantity (such as raised abdominal adiposity), a modified lipid profile, and the onset of resistance to insulin. It is stated in long-term research that menopausal changes are marked by a transition from a state that is regulated by high levels of estrogen to a state of high levels of androgen owing to an increase in bioavailable testosterone [29]. Several years after menopause, ovaries keep on releasing significant quantities of androgens, and elevated gonadotropin levels stimulate androgen secretion from ovaries despite low levels of estrogen. Sex-hormone-binding globulin levels decline as estrogen levels fall, resulting in a higher free androgen index and a reinforcement of the estrogen-androgen imbalance. An increase in bioavailable testosterone can cause visceral preadipocytes to accumulate fat. Estrogen inhibits the expression of androgen receptors in visceral adipocytes, leading to their increased expression. As a result of a reduction in levels of estrogen, visceral adiposity may become more prone to the negative consequences of androgens [30].

Moreover, variations in testosterone's bioavailability seem to predict visceral fat accumulation over five years. Also, the chances of obesity are greater in postmenopausal women whose amount of bioavailable testosterone rises faster compared to those whose amount stays the same. Women whose bioavailable testosterone levels rise more slowly, on the other hand, gain less fat. Because FSH receptors are found in visceral adipocytes, high amounts of FSH may also be a factor. Studies on animals have shown that FSH receptor signalling can boost the concentration of leptin and adipocyte lipid production while lowering adiponectin in serum; this makes it easier for fat to build up [31]. MetS is more likely to happen to women after menopause, in part because of the changes in hormones and metabolism that happen during this time. In this group, it is essential to fight the parts of MetS with the right lifestyle changes, like exercise. Different things around the world affect how common this syndrome is. These include location, ethnicity, gender, age, socioeconomic position, and education level. It is thought that insulin resistance ties all of these risk factors together. Even after taking into account age, it is thought that postmenopausal women are 1.6 times more prone to have MetS than younger women. So, menopause seems to play a unique role in why MetS happens in women. The development of metabolic abnormalities during the change from menopause to postmenopause has been mentioned as a critical intervention point for heart disease and other causes of death in women [27].

Cardiovascular System Changes

Cardiovascular ageing and disease are associated with an accelerated risk due to physical and physiological traits next to menopause, including dyslipidemia, resistance to insulin, redistribution of adipose tissue, and systemic hypertension. It seems that atherosclerosis is the culmination of cardiovascular-associated risks and their heightened expression during the perimenopausal stage. Further, endothelial dysfunction during menopause may be influenced by intricate relationships between oxidative stress, L-arginine, and asymmetric dimethylarginine levels. The overemphasized effects of altered cardiovascular system physiology, which affect peripheral, cardiac, and cerebrovascular beds, are the cause of the increased incidence of cardiovascular disorder associated with menopause. CVDs are the primary cause of mortality among women over the age of 50 worldwide, even more than breast or other female cancers. Traditional risk attributes of CVD consist of age, smoking, a sedentary lifestyle, eating poorly, elevated blood pressure, elevated body mass index, diabetes mellitus, cholesterol, or a history of early CVD in the family [32].

Males and females demonstrate variability in their risks for CVD. For example, women have a lower threat of CVD before menopause than men of the same age. Research supports that the decline in oestrogen production has been shown to increase the risk of coronary artery disease (CAD) [33]. After postmenopause, there is an apparent rise in CAD in women, which usually happens 10 years later [33]. It has been proposed that declining estradiol is the primary factor contributing to menopausal women's increased risk of cardiometabolic disease because of the sudden drop in estradiol levels along with modifications in the estrogen-to-androgen ratio that takes place at the time of menopause and is linked to a higher likelihood of CVD and type 2 diabetes [33]. Males and females demonstrate variability in their risks of CVD.

Obesity and Related Disorders

Adipocyte growth and the distribution of body fat are significantly influenced by sex hormones. Although testosterone and oestrogens have differing effects on adipocyte physiological processes, there is disagreement about the role that oestrogens play in the emergence of metabolic disorders during menopause. One crucial element in the increased risk of cardiovascular illness during postmenopause is the cessation of oestrogen production, which adversely affects lipid production and increases the risk of abdominal adipose accumulation. Sex steroids are needed to control how adipocytes use energy and to affect how specific fat stores change based on a person's gender [7]. In humans, the concentrations of sex hormones affect some of the factors that decide where fat is stored. Men have a lower average body fat than women but higher intra-abdominal/central adipose tissue. On the other hand, women possess higher levels of total body fat, most of it being in the gluteal/femoral and subcutaneous areas. Females deposit adipose in the lower abdomen when they have high levels of oestrogen production or take high amounts of exogenous estradiol, but when they stop making estrogen (and do not take it), they lose muscle mass and increase adipose around the waist, which makes them have a higher risk of CVD [28].

Thyroid Changes

The thyroid is a vital part of how metabolism works in our body. Thyroid hormones affect how glucose and fats are used, how blood pressure is controlled, and how much energy is used. Recent studies have shown that people with hypothyroidism or mild hypothyroidism are more likely to have MetS. Studies have shown that individuals whose thyroid stimulating hormone (TSH) levels were at the top of the usual spectrum (2.6-4.6 mU/L) were more prone to be obese, have higher triglyceride levels, and have MetS [34]. Regardless of whether their levels of TSH are under acceptable limits, young, healthy women with TSH values of more than 2.5 mU/L must be checked for MetS. Other studies didn't find a strong link between high levels of TSH and MetS. The thyroid is also affected by being overweight. A meta-analysis of research found a noteworthy link between obesity and a higher incidence of hypothyroidism (risk ratio (RR) = 1.86). Overweight people also had higher chances of having both overt and subclinical hypothyroidism (RR = 3.20 and RR = 1.71, respectively). Further study is warranted to include a larger population of females that have obesity and hypothyroidism which started after menopause [35].

Osteoporosis

Osteoporosis after menopause increases the risk of fractures and poor health outcomes. This change is a significant cause of a lower standard of daily life in menopausal women because it makes them more likely to experience bone fractures. It is also a significant health risk. At menopause, when there isn't enough oestrogen, the osteogenesis cycle is disrupted because resorption by osteoclasts increases lacking an increase in the activity of osteoblasts. This means that more bone is taken away than is put in, so there is a reduction in bone mass. At first, this process was called uncoupling. We know much about what happens to cells when there isn't enough oestrogen [36]. Tumour necrosis factor (TNF) production increases and stromal/osteoblastic lineage cells grow even more susceptible to IL-1. TNF and IL-1 cause the secretion of IL-6, macrophage colony-stimulating factor (M-CSF), IL-11, granulocyte M-CSF, and tumour growth factor by stromal cells and preosteoblasts. The receptor activator of nuclear factor B ligand (RANKL) is the last cytokine in osteoclastogenesis. Osteoblasts make it and bind to the RANK receptor on osteoclasts. Osteoprotegerin (OPG), a soluble receptor made by stromal osteoblast lineage cells, is RANKL's natural blocker. Oestrogen makes OPG work more. In hindsight, we are now aware that the particular uncoupling factor produced by osteoblasts is known as RANKL [36]. By making the number of pre-osteoclasts in the bone marrow pool bigger, these factors speed up bone loss. Oestrogen slows down these factors. The most crucial thing oestrogen does is make more OPG and less M-CSF and RANKL [22].

Liver Diseases

During menopause, there are a lot of physical and chemical changes that can affect the way the liver works and lead to liver disease. Menopause is characterized by increasing oestrogen deficiency, and oestrogen deficiency in physiologic ageing enhances the risk of impaired mitochondrial function, ageing of cells, decreased sensitivities of the immune system to infection, and an alteration in the equilibrium between oxidative stress and the synthesis of antioxidants. The development and progression of liver diseases are influenced by age and hormonal causes, making liver disease distinct. Research has shown that the combination of these elements has an adverse effect on the liver's overall wellness in postmenopausal women [37]. All of these changes can make people more likely to get serious hepatic disorders, such as nonalcoholic fatty liver disease and hepatocellular carcinoma. They can also hasten the progression of fibrosis in patients with hepatic disorders, which has been shown in hepatitis C virus liver disease in particular. The distinctiveness of such moderating elements must make perimenopausal and menopausal women more likely to have liver disease and give them a chance to get aggressive treatment so that liver disease doesn't get worse and lead to cirrhosis, liver cancer, or liver failure. These characteristics are expected to develop in the liver during menopause because the liver undergoes several morphologic changes as it ages. Decreases in the liver's blood volume and flow and modifications to its ability to regenerate the liver are examples of these changes [26,37].

*Menopause With Diabetes* There is a wealth of information in both the polycystic ovary syndrome (PCOS) and infertility literature that shows the significance of controlling insulin resistance as it adversely affects sex steroids. Diabetes mellitus, particularly type 2 diabetes mellitus (T2DM), more common during the menopausal transition due to increased rates of resistance to insulin and upper body fatty tissue accumulation, is predisposed to as a result. Additionally, type 1 and early-onset T2DM patients may have menopause sooner than people who don't have diabetes mellitus due to how the disease affects ovarian ageing [38].

Psychosexual Effect of Menopause

Oestrogens, androgens, and progesterone are ovarian hormones that have a variety of impacts on the neurological system. Through androgen-specific receptors and the aromatization of testosterone to estradiol, androgens' effects on the neural system are controlled [10]. Both androgen-specific receptors and the aromatization of testosterone to estradiol mediate the effects of androgens in the brain. Changes in the levels of androgens in the blood are crucial for the psychological and sexual transformations that follow menopause. Short-term estrogen therapy has been shown to improve postmenopausal women's emotional signs, maintain vaginal lubricity, minimize vaginal muscle atrophy, and improve blood flow to the pelvis. To address diminished sexual interest, psychological problems, or other menopausal issues related to sexuality, some patients need more than just estrogen. The results of experimental research show that hormone replacement treatment with oestrogen and androgens is effective in treating naturally menopausal women as well as those who undergo surgery and improves psychologic (e.g., depressive disorders, fatigue, and lack of concentration) and sexual (e.g., diminished libido and being unable to experience pleasure) symptoms more than oestrogen alone. Further, the use of vaginal oestrogen has been shown to be very effective for genitourinary symptoms of menopause such as urinary leakage, vaginal dryness and vaginal atrophy, without the cardiovascular and cancer risks of systemic oestrogen replacement [39].

Management

There is enough proof that vitamin D is suitable for the condition of paediatric and geriatric bone, but the same can't be said for females during their reproductive years. New evidence shows the significance of maintaining normal levels of vitamin D among people in general. This could help avoid long-term health problems like heart attacks, MetS, cancer, anxiety, depression, and even death. Interestingly, cardio exercise has been suggested as an essential add-on therapy for preventing and treating cardiometabolic diseases [40]. Our group has shown that aerobic exercise training in ovariectomized rats with naturally high blood pressure slowed or stopped the rise in triglycerides and glucose levels in the blood, decreased resistance to insulin caused by too much fructose, and enhanced heart disease autonomic modulation in these rats [41]. Recent clinical guidelines have endorsed resistance exercise training as an adjunct to aerobic activity (combined training) as an efficient non-pharmacological treatment method for cardiovascular and metabolic illnesses [40]. However, further research is needed to understand how combined exercise training affects oxidative stress, inflammation, and cardiovascular function. Thus, our investigation aimed to test the theory that, in hypertensive rats receiving deprivation of ovarian hormones, combined exercise training may lessen or even reverse the harm caused by fructose overload to the cardiovascular and renal systems. As a result, this research aimed to analyze the effects of varied physical activity on cardiovascular autonomic regulation and inflammation [41].

Hormone Replacement Therapy

In clinical practice, hormone replacement treatment (HRT) has been applied to postmenopausal women for many years. The advantages and disadvantages of HRT have been frequently confirmed and addressed with additional research and more recent studies. To alleviate vasomotor symptoms, prevent osteoporosis, and treat the genitourinary syndrome of menopause (GSM), HRT is advised. However, there is disagreement on the specific relationship between HRT and the threat of heart disease, venous thromboembolism, neurological disorders, breast cancer, and endometrial cancer [36]. Therefore, the initiation time, regimen, and duration of HRT must be altered to maximise benefits and minimise hazards. Recent research has shown that although HT could raise the frequency of several chronic illnesses, it is not linked to the chance of dying from any cause, heart disease, or breast cancer [26]. Early beginning HRT in symptomatic women under 60 has been shown to be significantly less risky for the development of cardiovascular and breast cancer. It is more likely that starting HRT near menopause at the lowest effective dose will have the most benefits and the fewest side effects. Although oral HRT formulations appear to provide additional therapeutic benefits in treating vasomotor symptoms and preventing osteoporosis, they have been shown overwhelmingly to have local effects only and do not increase the risks of circulating oestrogen as oral and transdermal formulations. You can assess your risk using the pooled cohort risk calculation for heart problems caused by atherosclerosis [26].

Summary of findings 

Menopause, or the cessation of menstrual periods, is brought on by a reduction in the hormones oestrogen and progesterone released by the ovaries. Menopause is an unavoidable phase for females; nevertheless, because each woman's experience is distinct, some need medical assistance to cope with the signs and symptoms. The physiological modifications that occur at various stages of the reproductive life cycle, as well as the kind and timing of menopause, generally contribute to an increased risk of cardiometabolic disease [42]. The most researched associations between menopause and cardiometabolic health are decreased ovarian production of oestrogen and relative androgen excess around the onset of menopause. Although testosterone and oestrogens have differing effects on adipocyte physiology, it is debatable how essential oestrogens are for the emergence of metabolic disorders following menopause.

The control of adipocyte differentiation by the central nervous system and the potential roles of estrogen-like substances and endocrine disruptor chemicals are reviewed in this systematic review of the subject. In general, men had a higher frequency of MetS than women. Female metabolism was significantly impacted by overt hyperthyroidism and subclinical hypothyroidism. Osteoporosis is another medical condition that menopausal women may experience [43]. Oestrogen deprivation is the main contributor to osteoporosis in menopausal women. The regular cycle of bone turnover is disrupted by the decrease in oestrogen secretion, which boosts osteoclastic degradation activity while decreasing osteoblastic activity. The entire article assesses and provides information on all the changes in a woman's life after menopause-adjunctive therapy for diagnosing, treating, and preventing cardiometabolic diseases. Our team has shown that aerobic exercise training in naturally hypertensive ovariectomized rats decreased insulin resistance brought on by fructose overload, attenuated and prevented blood glucose and triglyceride concentration increases, and also improved cardiovascular autonomic control in this condition [44].

Recent clinical guidelines have endorsed resistance exercise training as an adjunct to aerobic exercise (combined training) as an efficient non-pharmacological treatment method for cardiovascular and metabolic illnesses. However, there are many studies that support the importance of resistance training in postmenopause because ageing along with oestrogen depletion causes the deterioration of muscle, which is essential for optimum musculoskeletal function and metabolic rate, inflammation, and cardiovascular function [44]. A summary of the articles is tabulated in Table 1.

**Table 1:** Summary of the articles included in the review CVD: Cardiovascular diseases; GUS: Genitourinary syndrome; T2DM: Type 2 diabetes mellitus; HRT: Hormonal replacement therapy; HT: Hormonal therapy; MT: Menopausal transition; FSH: Follicle-stimulating hormone; LH: Luteinizing hormone; BED: Binge eating disorder; PCOS: Polycystic ovarian syndrome

Authors	Year	Findings
Nelson et al. [1]	2000	Menopause, which is characterized by decreased ovarian hormone release of progesterone and oestrogen, is the stage of life when menstrual cycles stop.
Innes et al. [2]	2008	The risk of developing CVD increases significantly with menopause. This is probably because atherogenic changes and an increase in insulin resistance coincide and combine to form the metabolic or insulin resistance syndrome, a collection of hemodynamic and metabolic abnormalities that are strongly linked to the development and course of cardiovascular disease.
Ambikairajah et al. [3]	2022	Coining and introduction of menopause.
Ko et al. [4]	2020	Menopause occurs in women as they age. Because of the decreased release of estrogen, menopause may cause a variety of alterations in lipid metabolism. These alterations impact the basal metabolic rate and include increased fat mass and decreased fat-free mass.
Hyvarinen et al. [5]	2022	After menopause, women’s metabolic health declines, and it has been generally suggested that physical activity can enhance cardiovascular health and the metabolic risk factor profile.
Phillips et al. [6]	2021	GUS of menopause is a group of symptoms that can affect postmenopausal women and are caused by decreased hormonal stimulation, particularly oestrogenic stimulation, to the lower urinary tract or vulvovaginal area.
Lambrinoudaki et al. [7]	2022	As menopause increases the likelihood of upper body adipose tissue buildup and the prevalence of insulin resistance, the menopause transition is accompanied by metabolic alterations predisposing to diabetes mellitus, particularly T2DM.
Monteleone et al. [8]	2018	Menopausal symptoms significantly impact women’s quality of life and productivity at work; developing methods of coping and raising awareness of the symptoms may be helpful.
Kontis et al. [9]	2017	Forecasts of mortality and life expectancy in the future.
Bermingham et al. [10]	2022	Unfavourable changes in body composition, fasting, postprandial profiles (including postprandial glycemia and inflammation), nutrition, sleep patterns, and gut flora are linked to postmenopausal status.
Mehta et al. [11]	2021	The most successful treatment for the common and negatively affecting vasomotor symptoms of menopause is HT.
Kim et al. [12]	2019	The advantages, disadvantages, and recent methods of management in the HT of menopause.
Talaulikar et al. [13]	2022	The time interval between the onset of irregular monthly cycles, which are typically accompanied by some menopausal symptoms, and menopause is known as the menopausal transition.
O’Neill et al. [14]	2017	HRT is commonly utilized to treat symptoms of oestrogen withdrawal, including vaginal dryness, hot flashes, dyspareunia, and night sweats. However, HRT has not been shown to be effective in treating arthritis or midlife depression.
Dennerstein et al. [15]	2002	The MT is accompanied by a decrease in sexual functioning. And there is a strong correlation between deteriorating sexual function and estradiol.
Sherman et al. [16]	1975	Lower estradiol levels and elevated FSH concentrations were observed in the few cycles preceding and amidst the menopausal transition, with LH levels remaining within the normal range.
Santoro et al. [17]	2004	A number of dynamic changes in physiology occur throughout the menopause transition stage of life.
Santora et al. [18]	1996	Hypergonadotropism, hyperestrogenism, and reduced luteal phase progesterone are examples of the altered ovarian function associated with perimenopause, which can be seen as early as age 43. This stage of life is marked by increased gynaecological morbidity, which could very likely be caused by these hormonal changes.
Burger et al. [19]	2008	Late reproductive age tends to predispose to irregular and unpredictable cycle characteristics due to a large decline in follicle counts. Measuring FSH, or estradiol, in an attempt to stage a person in relation to approaching menopause, but it is unreliable because there is no particular endocrine marker of early or late menopause.
Rothman et al. [20]	2011	Researchers and medical professionals have faced a technological hurdle when using immunoassays to assess sex steroid hormones.
Lizcano et al. [21]	2014	Multiple elements of glucose and lipid metabolism are regulated by estrogens and estrogen receptors. Women who have disruptions to these metabolic signals are more likely to develop metabolic syndrome and have increased cardiovascular risks.
Mumusoglu et al. [22]	2019	In older women, the menopause itself raises the risk of metabolic syndrome. Menopause may have an impact on certain aspects of metabolic syndrome, such as insulin resistance, hypertension, central obesity, and dyslipidemia.
Jeong et al. [23]	2022	Since metabolic syndrome and non-alcoholic liver disease raise the risk of cardiovascular events and result in long-term health issues in middle-aged women who live 30–40 years following menopause, their significance is further highlighted.
Refaei et al. [24]	2022	Women going through menopause may experience tremendous feelings of confidence and fear about the future and its ramifications. Women ask their relatives, friends, and healthcare practitioners for solutions to these issues.
Santoro et al. [25]	2015	Significant symptoms are linked to the menopausal transition and the postmenopausal years.
Luoto et al. [26]	2009	The prolonged perimenopausal phase and early menopausal start are risk factors for declining well-being during menopause. High body mass index and smoking are additional risk factors for an increased frequency of hot flashes.
Tran et al. [27]	2023	Depending on menopausal status, alterations in metabolic syndrome are linked to an increased risk of endometrial and breast cancer in the future.
Hidalgo et al. [28]	2020	It has been suggested that changing one’s lifestyle is the best prevention against metabolic syndrome. The two most commonly promoted therapies are physical activity and a balanced diet.
Udo et al. [29]	2014	We discovered that postmenopausal women had a higher likelihood than premenopausal women of having a clinically increased level of total cholesterol and impaired glycemic control among obese patients with BED.
Marsh et al. [30]	2023	Thus, exercise continues to be the most effective treatment agent for reducing metabolic dysfunction linked to menopause and is an essential behavioural strategy to prevent and ameliorate health deterioration in this population.
Nair et al. [31]	2021	The main points of this review are the differing impacts of menopause on cardiovascular disease at the subclinical, biochemical, and molecular levels.
Roa-Diaz et al. [32]	2021	In this review, we outline the key factors that raise the risk of cardiometabolic disease in older women and offer suggestions for further study.
Yin et al. [33]	2017	Thyroid function and metabolic alterations.
He et al. [34]	2021	Co-relationships in thyroxine level and metabolic X syndrome.
Tella et al. [35]	2014	For women who experience menopausal symptoms (such as hot flashes and vasomotor symptoms) within 10 years of menopause, oestrogen therapy and hormone therapy may be the primary treatments to avoid fractures and bone loss.
Brady et al. [36]	2015	The development and course of liver disease in postmenopausal women are specifically influenced by the combination of age and hormonal variables.
Sarrel et al. [37]	1999	It has been said that the absence and withdrawal of ovarian hormones affect the brain. It seems that androgens have a significant influence on women’s psychophysiology both before and after menopause.
Conti et al. [38]	2015	The results of this study show that ovarian hormone deprivation increased impairment in parameters related to metabolism, cardiovascular disease, inflammation, the autonomic nervous system, and oxidative stress and that coupled exercise training was useful in mitigating or returning these illnesses to normal.
Dadoniene et al. [39]	2018	Vitamin D deficiency was present in a large percentage of postmenopausal metabolic women. However, neither vitamin D nor bone health were correlated.
Pan et al. [40]	2022	Early hormone therapy in symptomatic postmenopausal women under 60 years of age who do not have any contraindications is safe and likely to reduce mortality over time.
Gu et al. [41]	2022	Female subjects at reproductive age are affected by PCOS, a prevalent endocrine and metabolic condition.
Ko et al. [42]	2021	Due to a decrease in estradiol release, both natural and surgical menopause are associated with alterations in body composition as well as potential modifications to lipid and energy metabolism.
Son et al. [43]	2021	Training with resistance bands reduces the advancement of metabolic syndrome in obese postmenopausal women.
Baccaro et al. [44]	2015	Postmenopausal osteoporosis increases the risk of fracture and has a detrimental effect on older women’s health.

## Conclusions

This narrative review, which explored the endocrine changes in postmenopausal women, has provided a comprehensive overview of the intricate hormonal shifts that mark this significant life stage. The journey through menopause is accompanied by a complex interplay of hormonal changes, including the decline in oestrogen and progesterone production, leading to physiological and metabolic transformations. During the course of the research, the authors have considered how these hormone fluctuations affect women's health in various ways, from bone mass and cardiovascular wellness to cognition and mood. The results of this study have shown an increased likelihood of developing illnesses, including bone loss, CVD, and a metabolic disorder, and it highlights the significance of individualised healthcare approaches to lessen these possible effects. It has been suggested that HRT, along with various therapies, could be used to alleviate some of the adverse effects. Still, their implications and long-term impacts need to be carefully considered. The review also clarified the impact of lifestyle factors on postmenopausal women's hormone balance and general well-being, such as the importance of food, exercise, and stress management. These insights underscore the significance of holistic approaches to health maintenance during and after the transition into menopause. By addressing the challenges of endocrine changes, healthcare professionals can empower postmenopausal women to embrace this new phase of life with vitality and confidence.
